# Global dynamics for an SIR patchy model with susceptibles dispersal

**DOI:** 10.1186/1687-1847-2012-131

**Published:** 2012-08-01

**Authors:** Luju Liu, Weiyun Cai, Yusen Wu

**Affiliations:** School of Mathematics and Statistics, Henan University of Science and Technology, Luoyang, 471003 P.R. China

**Keywords:** H1N1 Influenza, Epidemic Model, Comparison Principle, Endemic Equilibrium, Basic Reproduction Number

## Abstract

An SIR epidemiological model with suscptibles dispersal between two patches is addressed and discussed. The basic reproduction numbers $R_{01}$ and $R_{02}$ are defined as the threshold parameters. It shows that if both $R_{01}$ and $R_{02}$ are below unity, the disease-free equilibrium is shown to be globally asymptotically stable by using the comparison principle of the cooperative systems. If $R_{01}$ is above unity and $R_{02}$ is below unity, the disease persists in the first patch provided $S_{2}^{1*}<S_{2}^{2*}$. If $R_{02}$ is above unity, $R_{01}$ is below unity, and $S_{1}^{2*}<S_{1}^{1*}$, the disease persists in the second patch. And if $R_{01}$ and $R_{02}$ are above unity, and further $S_{2}^{1*}>S_{2}^{2*}$ and $S_{1}^{2*}>S_{1}^{1*}$ are satisfied, the unique endemic equilibrium is globally asymptotically stable by constructing the Lyapunov function. Furthermore, it follows that the susceptibles dispersal in the population does not alter the qualitative behavior of the epidemiological model.

**Electronic supplementary material:**

The online version of this article (doi:10.1186/1687-1847-2012-131) contains supplementary material, which is available to authorized users.

## 1 Introduction

The development of economic globalization and the progression of science and technology yield more and more frequent contact and communication between people in different countries and regions, which further directly accelerates the development of global economy and fosters the prosperity and flourishing of a society. However, the bad things may occur simultaneously, such as, the spread of 2003 SARS and 2009 H1N1 influenza almost throughout the world. SARS involved 30 countries and regions, caused more than 8,000 patients, and 774 deaths [19, 20]. The H1N1 influenza virus quickly spread worldwide due to airplane travel. As of May 6, 2009, the virus had invaded in 23 countries including Mexico and the United States, and a total of 1,882 people were confirmed to be infected by it [3]. It then follows that the studies on the influence of infectious diseases transmission on the global population that formulates patchy models are more and more significant and practical.

A great number of mathematical patchy models have been proposed and analyzed to illustrate the influence of the transmission of infectious diseases on the local population among many countries and regions [1, 2, 7, 12, 18]. But for many mathematical models of infectious diseases in a patchy environment, the global stability of the endemic equilibrium is still an open problem. Motivated by this, in the present paper, a class of simple SIR models with susceptibles dispersal in a patchy environment is to be formulated and investigated the stability of the endemic equilibrium by constructing the Lyapunov function (also see [5, 6, 9–11, 13, 14]).

The rest of this paper is organized as follows. In Sect. 2, the SIR model with susceptibles dispersal between two disjoint patches is formulated, and the existence, uniqueness, and boundedness of the solutions are analyzed. The existence of equilibria and the basic reproduction numbers are derived in Sect. 3. In Sect. 4, the long-term behavior of the SIR model is studied. The brief conclusions and discussions are given in Sect. 5.

## 2 Model formulation

**Figure 1:**
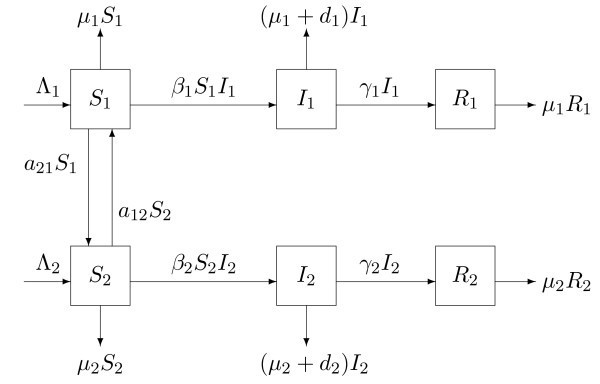
**The transfer diagram of a class of** SIR **epidemic models.**

In this section, a class of SIR epidemic models for infectious diseases between two patches is developed, in which only susceptible people may disperse between two disjoint patches. All the persons are classified into three compartments: susceptible (*S*), infectious (*I*), and removed (*R*) in each patch, respectively. It is assumed that the mass action incidence is used and there is no birth or death during travel. Based on the transfer diagram of Figure 1, the SIR epidemic model to understand the impact of susceptibles dispersal on the whole population is described by the following system of ordinary differential equations:

1 $$\begin{array}{r} \frac{dS_{1}}{dt}=\Lambda_{1}-\beta_{1}S_{1}I_{1}-\mu_{1}S_{1}+a_{12}S_{2}-a_{21}S_{1},\\ \frac{dI_{1}}{dt}=\beta_{1}S_{1}I_{1}-(\mu_{1}+d_{1}+\gamma_{1})I_{1},\\ \frac{dS_{2}}{dt}=\Lambda_{2}-\beta_{2}S_{2}I_{2}-\mu_{2}S_{2}+a_{21}S_{1}-a_{12}S_{2},\\ \frac{dI_{2}}{dt}=\beta_{2}S_{2}I_{2}-(\mu_{2}+d_{2}+\gamma_{2})I_{2}. \end{array}$$

Since $R_{1}$ and $R_{2}$ do not involve in other equations but themselves in system (1), they are not directly taken into account in system (1).

$\Lambda_{i}$ (i=1,2) is the recruitment constant rate of the population in the *i* th patch. $\beta_{i}$ (i=1,2) represents the transmission rate in the *i* th patch. $\mu_{i}$ (i=1,2) represents the natural death rate in the *i* th patch. $d_{i}$ (i=1,2) is the induced-death rate in the *i* th patch. $\gamma_{i}$ (i=1,2) is the recovery rate of the infectious persons in the *i* th patch. $a_{12}$ represents the dispersal rate of susceptible individuals from the second patch to the first patch. $a_{21}$ represents the dispersal rate of susceptible individuals from the first patch to the second patch. All the parameters considered in the present paper are nonnegative. $N_{i}(t)$ (i=1,2) denotes the number of the total population in the *i* th patch at time *t*. Therefore, $N_{i}=S_{i}+I_{i}+R_{i}$ (i=1,2).

By applying the Theorem 5.2.1 of [15], it then follows that for any $(S_{10},I_{10},S_{20},I_{20})\in R_{+}^{4}$, system (1) exists a unique local nonnegative solution $\left(S_{1}(t),I_{1}(t),S_{2}(t),I_{2}(t)\right)$ through the initial value $\left(S_{1}(0),I_{1}(0),S_{2}(0),I_{2}(0))=(S_{10},I_{10},S_{20},I_{20}\right)$.

The expressions of $N_{i}$ and Eq. (1) give rise to the following formula:

2 $$\begin{array}{rcl} \frac{dN_{1}}{dt}+\frac{dN_{2}}{dt} & = & \Lambda_{1}+\Lambda_{2}-\mu_{1}N_{1}-\mu_{2}N_{2}-d_{1}I_{1}-d_{2}I_{2}\\ \leq & \Lambda_{1}+\Lambda_{2}-\min\{\mu_{1},\mu_{2}\}(N_{1}+N_{2}). \end{array}$$

System (2) implies ${lim sup}_{t\to \infty }(N_{1}+N_{2})\leq (\Lambda_{1}+\Lambda_{2})/\min\{\mu_{1},\mu_{2}\}$. Therefore, $N_{1}+N_{2}$ is ultimately bounded and all the solutions of system (1) globally exists on the interval [0,∞). The aforementioned discussions can be summarized into the following results.

**Theorem 2.1** *System* (1) *exists a unique and bounded solution throughout the initial value* $(S_{10},I_{10},S_{20},I_{20})\in R_{+}^{4}$. *Further*, *the compact set*

$$\Omega :=\left\{(S_{1},I_{1},S_{1},I_{2})\in R_{+}^{4}:S_{1}+I_{1}+S_{1}+I_{2}\leq \frac{\Lambda_{1}+\Lambda_{2}}{\min\{\mu_{1},\mu_{2}\}}\right\}$$

*is a positively invariant set and attracts all positive orbits in* $R_{+}^{4}$.

Note that the long-time behaviors of the solutions of system (1) are investigated in region Ω instead of the space $R_{+}^{4}$.

## 3 Equilibria and the basic reproduction numbers

In this section, the existence of equilibria and the basic reproduction numbers are studied. By the direct calculation, system (1) always exhibits one disease-free equilibrium $P_{0}=(S_{1}^{0},0,S_{2}^{0},0)$ for all parameters, where

$$S_{1}^{0}=\frac{\Lambda_{1}a_{12}+\Lambda_{2}a_{12}+\Lambda_{1}\mu_{2}}{\mu_{1}\mu_{2}+\mu_{2}a_{21}+\mu_{1}a_{12}},\;S_{2}^{0}=\frac{\Lambda_{2}a_{21}+\Lambda_{1}a_{21}+\Lambda_{2}\mu_{1}}{\mu_{1}\mu_{2}+\mu_{2}a_{21}+\mu_{1}a_{12}}.$$

Applying the next generation matrix approach developed in [4] gives rise to the following formulas:

$$F=[\begin{array}{cc} \beta_{1}S_{1}^{0} & 0\\ 0 & \beta_{2}S_{2}^{0} \end{array}]=:[\begin{array}{cc} F_{1} & 0\\ 0 & F_{2} \end{array}],$$

and

$$V=[\begin{array}{cc} \mu_{1}+d_{1}+\gamma_{1} & 0\\ 0 & \mu_{2}+d_{2}+\gamma_{2} \end{array}]=:[\begin{array}{cc} V_{1} & 0\\ 0 & V_{2} \end{array}].$$

Therefore, the basic reproduction number is defined as

$$\begin{array}{rcl} R_{0} & = & \rho \left(FV^{-1}\right)=\max\left\{\rho \left(F_{1}V_{1}^{-1}\right),\rho \left(F_{2}V_{2}^{-1}\right)\right\}\\ = & \max\{R_{01},R_{02}\}=\max\left\{\frac{\beta_{1}S_{1}^{0}}{\mu_{1}+d_{1}+\gamma_{1}},\frac{\beta_{2}S_{2}^{0}}{\mu_{2}+d_{2}+\gamma_{2}}\right\}, \end{array}$$

where ρ(M) denotes for the spectral radius of the matrix **M**, $R_{01}$ and $R_{02}$ correspond to the basic reproduction numbers of the first and the second patch when there is no dispersal between two patches, respectively. The proof process of [4], Theorem 2 implies the following statements.

**Lemma 3.1** *There hold*

1. *Let* $M_{1}=F_{1}-V_{1}$ *and* $s(M_{1})$ *be the maximum real part of all the eigenvalues of the matrix* $M_{1}$. *Then* $s(M_{1})<0$ *if and only if* $R_{01}<1$, *and* $s(M_{1})>0$ *if and only if* $R_{01}>1$;
2. *Let* $M_{2}=F_{2}-V_{2}$ *and* $s(M_{2})$ *be the maximum real part of all the eigenvalues of the matrix* $M_{2}$. *Then* $s(M_{2})<0$ *if and only if* $R_{02}<1$, *and* $s(M_{2})>0$ *if and only if* $R_{02}>1$.

Furthermore, if $R_{01}>1$ and $R_{02}<1$, there exists a nontrivial boundary equilibrium $P_{1*}=(S_{1}^{1*},I_{1}^{1*},S_{2}^{1*},0)$, where 

If $R_{02}>1$ and $R_{01}<1$, there exists another nontrivial boundary equilibrium $P_{2*}=(S_{1}^{2*},0,S_{2}^{2*},I_{2}^{2*})$, where 

If $R_{01}>1$, $R_{02}>1$, $S_{1}^{2*}>S_{1}^{1*}$, and $S_{2}^{1*}>S_{2}^{2*}$, system (1) admits exactly one endemic equilibrium $P_{**}=(S_{1}^{**},I_{1}^{**},S_{2}^{**},I_{2}^{**})$, where

$$S_{1}^{**}=S_{1}^{1*},\;I_{1}^{**}=\frac{S_{1}^{2*}-S_{1}^{**}}{(\mu_{1}+a_{21})\beta_{1}S_{1}^{**}},\;S_{2}^{**}=S_{2}^{2*},\;I_{2}^{**}=\frac{S_{2}^{1*}-S_{2}^{**}}{(\mu_{2}+a_{12})\beta_{2}S_{2}^{**}}.$$

## 4 Threshold dynamics

In this section, the stability of equilibria is to be formulated. First of all, the global stability of the disease-free equilibrium $P_{0}$ is to be discussed. There holds the following result.

**Theorem 4.1** *If the basic reproduction number* $R_{0}$ *is less than one*, *the disease*-*free equilibrium* $P_{0}$ *is globally asymptotically stable*; *while if the basic reproduction number* $R_{0}$ *is greater than one*, *the disease*-*free equilibrium* $P_{0}$ *is unstable*.

*Proof* If $R_{0}<1$, [4], Theorem 2, yields that $P_{0}$ is locally asymptotically stable. Thus, it is sufficient to prove the global attractivity of $P_{0}$ when $R_{0}<1$. The first and third equations of system (1) implies 

It is easy to see the following linear system:

3 $$\begin{array}{r} \frac{d{\hat{S}}_{1}}{dt}=\Lambda_{1}-(\mu_{1}+a_{21}){\hat{S}}_{1}+a_{12}{\hat{S}}_{2},\\ \frac{d{\hat{S}}_{2}}{dt}=\Lambda_{2}-(\mu_{2}+a_{12}){\hat{S}}_{2}+a_{21}{\hat{S}}_{1}, \end{array}$$

has a positive equilibrium ${\hat{S}}_{0}=(S_{1}^{0},S_{2}^{0})$ and ${\hat{S}}_{0}$ is globally asymptotically stable for system (3) in $R_{+}^{2}$. Consequently, the comparison principle of cooperative systems [16], Theorem B.1, yields that for any ε>0, $S_{i}(t)<S_{i}^{0}+\varepsilon$ (i=1,2) is satisfied, for sufficiently large *t*. Thus, if *t* is sufficiently large, the second and fourth equations of system (1) admit 

Thus, it suffices to prove the following system:

4 $$\begin{array}{r} \frac{d{\tilde{I}}_{1}}{dt}=\left(\beta_{1}S_{1}^{0}-(\mu_{1}+d_{1}+\gamma_{1})\right){\tilde{I}}_{1}+\varepsilon \beta_{1}{\tilde{I}}_{1},\\ \frac{d{\tilde{I}}_{2}}{dt}=\left(\beta_{2}S_{2}^{0}-(\mu_{2}+d_{2}+\gamma_{2})\right){\tilde{I}}_{2}+\varepsilon \beta_{2}{\tilde{I}}_{2}, \end{array}$$

tends to the zero solution as *t* goes to infinity. Let ${\bar{M}}_{1}=\beta_{1}$, and ${\bar{M}}_{2}=\beta_{2}$. $R_{0}<1$ implies $R_{01}<1$ and $R_{02}<1$. Lemma 3.1 implies $s(M_{1})<0$ and $s(M_{2})<0$. By the continuity of $s(M_{1}+\varepsilon {\bar{M}}_{1})$ and $s(M_{2}+\varepsilon {\bar{M}}_{2})$ in *ε*, *ε* can be chosen small enough so that $s(M_{1}+\varepsilon {\bar{M}}_{1})<0$ and $s(M_{2}+\varepsilon {\bar{M}}_{2})<0$. Consequently, the solutions of system (4) approach to zero with *t* going to infinity. The comparison principle of cooperative systems [16], Theorem B.1, implies $I_{1}(t)\to 0$ and $I_{2}(t)\to 0$ as t→∞. Therefore, the theory of asymptotically autonomous systems [17], Theorem 1.2, shows that $\lim_{t\to \infty }S_{i}(t)=S_{i}^{0}$ (i=1,2).

In the case of $R_{0}>1$, [4], Theorem 2, admits that $P_{0}$ is unstable, which finishes the theorem. □

Next, the two results regarding the stability of the boundary equilibria are given by applying the so-called Routh-Hurwitz criterion.

**Theorem 4.2** *If* $R_{01}>1$ *and* $R_{02}<1$, *the boundary equilibrium* $P_{1*}$ *is stable when* $S_{2}^{1*}<S_{2}^{2*}$; *while the boundary equilibrium* $P_{1*}$ *is unstable when* $S_{2}^{1*}>S_{2}^{2*}$.

*Proof* $R_{0}>1$ and $R_{02}<1$ imply that system (1) has a boundary equilibrium $P_{1*}$. The Jacobian matrix of the right-hand side of system (1) at the equilibrium $P_{1*}$, ordering coordinates as $\left(S_{1},S_{2},I_{1},I_{2}\right)$, is given by

$$M(P_{1*})=[\begin{array}{cccc} -\beta_{1}I_{1}^{1*}-(\mu_{1}+a_{21}) & a_{12} & -\beta_{1}S_{1}^{1*} & 0\\ a_{21} & -(\mu_{2}+a_{12}) & 0 & -\beta_{2}S_{2}^{1*}\\ \beta_{1}I_{1}^{1*} & 0 & 0 & 0\\ 0 & 0 & 0 & \hat{b} \end{array}],$$

where $\hat{b}=\beta_{2}S_{2}^{1*}-(\mu_{2}+d_{2}+\gamma_{2})=\beta_{2}(S_{2}^{1*}-S_{2}^{2*})$. Therefore, the eigenvalues are: $\hat{b}$ and the solutions of the following cubic equation:

5 $$\lambda^{3}+b_{1}\lambda^{2}+b_{2}\lambda +b_{3}=0,$$

where 

Since

$$\begin{array}{rcl} b_{1}b_{2}-b_{3} & = & \left(\mu_{1}+a_{21}+\beta_{1}I_{1}^{1*}\right)b_{2}\\ +(\mu_{2}+a_{12})\left(\mu_{1}\mu_{2}+\mu_{2}a_{21}+\mu_{2}\beta_{1}I_{1}^{1*}+\mu_{1}a_{12}+a_{12}\beta_{1}I_{1}^{1*}\right)>0, \end{array}$$

Routh-Hurwitz criterion implies all the roots of Eq. (5) have a negative real part.

Therefore, $S_{2}^{1*}<S_{2}^{2*}$ yields the boundary equilibrium $P_{1*}$ is locally stable; while $S_{2}^{1*}>S_{2}^{2*}$ demonstrates the boundary equilibrium $P_{1*}$ is unstable. □

**Theorem 4.3** *If* $R_{02}>1$ *and* $R_{01}<1$, *the boundary equilibrium* $P_{2*}$ *is stable when* $S_{1}^{2*}<S_{1}^{1*}$; *while the boundary equilibrium* $P_{2*}$ *is unstable when* $S_{1}^{2*}>S_{1}^{1*}$.

*Proof* Because $R_{02}>1$ and $R_{01}<1$, there exists another boundary equilibrium $P_{2*}$ for system (1). The Jacobian matrix of the right-hand side of system (1) at the equilibrium $P_{2*}$ is denoted by

$$M(P_{2*})=[\begin{array}{cccc} -(\mu_{1}+a_{21}) & -\beta_{1}S_{1}^{2*} & a_{12} & 0\\ 0 & \hat{c} & 0 & 0\\ a_{21} & 0 & -\beta_{2}I_{2}^{2*}-(\mu_{2}+a_{12}) & -\beta_{2}S_{2}^{2*}\\ 0 & 0 & \beta_{2}I_{2}^{2*} & 0 \end{array}],$$

where $\hat{c}=\beta_{1}S_{1}^{2*}-(\mu_{1}+d_{1}+\gamma_{1})=\beta_{1}(S_{1}^{2*}-S_{1}^{1*})$.

It is easy to see that all the eigenvalues of the matrix $M(P_{2*})$ are: $\hat{c}$ and the roots of the following equation:

6 $$\lambda^{3}+c_{1}\lambda^{2}+c_{2}\lambda +c_{3}=0,$$

where 

Because

$$\begin{array}{rcl} c_{1}c_{2}-c_{3} & = & \left(\mu_{2}+a_{12}+\beta_{2}I_{2}^{2*}\right)c_{3}\\ +(\mu_{1}+a_{21})\left(\mu_{1}\mu_{2}+\mu_{1}a_{12}+\mu_{1}\beta_{2}I_{2}^{2*}+\mu_{2}a_{21}+a_{21}\beta_{2}I_{2}^{2*}\right)>0, \end{array}$$

by using the Routh-Hurwitz criterion, it then follows that the real part of all the solutions of (6) is negative.

Furthermore, it is easier to see that if $S_{1}^{2*}<S_{1}^{1*}$, the boundary equilibrium $P_{2*}$ is locally stable; while if $S_{1}^{2*}>S_{1}^{1*}$, the boundary equilibrium $P_{2*}$ is unstable. □

Now we are in the position to discuss the global stability of the endemic equilibrium.

**Theorem 4.4** *If the following statements hold*:

(i) $R_{01}>1$;
(ii) $R_{02}>1$;
(iii) $S_{1}^{2*}>S_{1}^{1*}$;
(iv) $S_{2}^{1*}>S_{2}^{2*}$;

*then the endemic equilibrium* $P_{**}$ *is globally asymptotically stable*.

*Proof* Conditions (i)-(iv) imply system (1) exists the endemic equilibrium $P_{**}$. Next, we study the stability of the endemic equilibrium $P_{**}$ by using the Lyapunov approach.

The following equations are derived at the endemic equilibrium $P_{**}$:

7 $$\begin{array}{r} \Lambda_{1}=\beta_{1}S_{1}^{**}I_{1}^{**}+\mu_{1}S_{1}^{**}-a_{12}S_{2}^{**}+a_{21}S_{1}^{**},\\ \mu_{1}+d_{1}+\gamma_{1}=\beta_{1}S_{1}^{**},\\ \Lambda_{2}=\beta_{2}S_{2}^{**}I_{2}^{**}+\mu_{2}S_{2}^{**}-a_{21}S_{1}^{**}+a_{12}S_{2}^{**},\\ \mu_{2}+d_{2}+\gamma_{2}=\beta_{2}S_{2}^{**}. \end{array}$$

Construct the following Lyapunov function:

8 $$U=S_{1}-S_{1}^{**}\ln S_{1}+I_{1}-I_{1}^{**}\ln I_{1}+A\left(S_{2}-S_{2}^{**}\ln S_{2}\right)+A\left(I_{2}-I_{2}^{**}\ln I_{2}\right),$$

where $A=\frac{a_{12}S_{2}^{**}}{a_{21}S_{1}^{**}}$.

Differentiating the function *V* along with the solutions of system (1) with respect to time *t* gives

$$\frac{dU}{dt}{|}_{\left(\text{1}\right)}=\left(1-\frac{S_{1}^{**}}{S_{1}}\right)\frac{dS_{1}}{dt}+\left(1-\frac{I_{1}^{**}}{I_{1}}\right)\frac{dI_{1}}{dt}+A\left(1-\frac{S_{2}^{**}}{S_{2}}\right)\frac{dS_{2}}{dt}+A\left(1-\frac{I_{2}^{**}}{I_{2}}\right)\frac{dI_{2}}{dt}.$$

Combining system (1) admits

$$\begin{array}{rcl} \frac{dU}{dt}{|}_{\left(\text{1}\right)} & = & \left(1-\frac{S_{1}^{**}}{S_{1}}\right)(\Lambda_{1}-\beta_{1}S_{1}I_{1}-\mu_{1}S_{1}+a_{12}S_{2}-a_{21}S_{1})\\ +\left(1-\frac{I_{1}^{**}}{I_{1}}\right)\left[\beta_{1}S_{1}I_{1}-(\mu_{1}+d_{1}+\gamma_{1})I_{1}\right]\\ +A\left(1-\frac{S_{2}^{**}}{S_{2}}\right)(\Lambda_{2}-\beta_{2}S_{2}I_{2}-\mu_{2}S_{2}+a_{21}S_{1}-a_{12}S_{2})\\ +A\left(1-\frac{I_{2}^{**}}{I_{2}}\right)\left[\beta_{2}S_{2}I_{2}-(\mu_{2}+d_{2}+\gamma_{2})I_{2}\right]. \end{array}$$

Applying Eq. (7) shows

$$\begin{array}{rcl} \frac{dU}{dt}{|}_{\left(\text{1}\right)} & = & \left(1-\frac{S_{1}^{**}}{S_{1}}\right)\left(\beta_{1}S_{1}^{**}I_{1}^{**}+\mu_{1}S_{1}^{**}-a_{12}S_{2}^{**}+a_{21}S_{1}^{**}-\beta_{1}S_{1}I_{1}-\mu_{1}S_{1}+a_{12}S_{2}-a_{21}S_{1}\right)\\ +A\left(1-\frac{S_{2}^{**}}{S_{2}}\right)(\beta_{2}S_{2}^{**}I_{2}^{**}+\mu_{2}S_{2}^{**}-a_{21}S_{1}^{**}+a_{12}S_{2}^{**}\\ -\beta_{2}S_{2}I_{2}-\mu_{2}S_{2}+a_{21}S_{1}-a_{12}S_{2})\\ +\left(1-\frac{I_{1}^{**}}{I_{1}}\right)\left(\beta_{1}S_{1}I_{1}-\beta_{1}S_{1}^{**}I_{1}\right)+A\left(1-\frac{I_{2}^{**}}{I_{2}}\right)\left(\beta_{2}S_{2}I_{2}-\beta_{2}S_{2}^{**}I_{2}\right). \end{array}$$

Rearranging the above equation, it then follows

$$\begin{array}{rcl} \frac{dU}{dt}{|}_{\left(\text{1}\right)} & = & \mu_{1}S_{1}^{**}\left(1-\frac{S_{1}^{**}}{S_{1}}\right)\left(1-\frac{S_{1}}{S_{1}^{**}}\right)+\beta_{1}S_{1}^{**}I_{1}^{**}\left(1-\frac{S_{1}^{**}}{S_{1}}\right)\left(1-\frac{S_{1}I_{1}}{S_{1}^{**}I_{1}^{**}}\right)\\ +a_{12}S_{2}^{**}\left(1-\frac{S_{1}^{**}}{S_{1}}\right)\left(\frac{S_{2}}{S_{2}^{**}}-1\right)+a_{21}S_{1}^{**}\left(1-\frac{S_{1}^{**}}{S_{1}}\right)\left(1-\frac{S_{1}}{S_{1}^{**}}\right)\\ +\beta_{1}S_{1}^{**}I_{1}^{**}\left(1-\frac{I_{1}^{**}}{I_{1}}\right)\left(\frac{S_{1}I_{1}}{S_{1}^{**}I_{1}^{**}}-\frac{I_{1}}{I_{1}^{**}}\right)+A\mu_{2}S_{2}^{**}\left(1-\frac{S_{2}^{**}}{S_{2}}\right)\left(1-\frac{S_{2}}{S_{2}^{**}}\right)\\ +A\beta_{2}S_{2}^{**}I_{2}^{**}\left(1-\frac{S_{2}^{**}}{S_{2}}\right)\left(1-\frac{S_{2}I_{2}}{S_{2}^{**}I_{2}^{**}}\right)+Aa_{21}S_{1}^{**}\left(1-\frac{S_{2}^{**}}{S_{2}}\right)\left(\frac{S_{1}}{S_{1}^{**}}-1\right)\\ +Aa_{12}S_{2}^{**}\left(1-\frac{S_{2}^{**}}{S_{2}}\right)\left(1-\frac{S_{2}}{S_{2}^{**}}\right)+A\beta_{2}S_{2}^{**}I_{2}^{**}\left(1-\frac{I_{2}^{**}}{I_{2}}\right)\left(\frac{S_{2}I_{2}}{S_{2}^{**}I_{2}^{**}}-\frac{I_{2}}{I_{2}^{**}}\right). \end{array}$$

By denoting $x:=\frac{S_{1}}{S_{1}^{**}}$, $y:=\frac{I_{1}}{I_{1}^{**}}$, $z:=\frac{S_{2}}{S_{2}^{**}}$, and $w:=\frac{I_{2}}{I_{2}^{**}}$, the above formula can be rewritten as

$$\begin{array}{rcl} \frac{dU}{dt}{|}_{\left(\text{1}\right)} & = & \mu_{1}S_{1}^{**}\left(1-\frac{1}{x}\right)(1-x)+\beta_{1}S_{1}^{**}I_{1}^{**}\left(1-\frac{1}{x}\right)(1-xy)+a_{12}S_{2}^{**}\left(1-\frac{1}{x}\right)(z-1)\\ +a_{21}S_{1}^{**}\left(1-\frac{1}{x}\right)(1-x)+\beta_{1}S_{1}^{**}I_{1}^{**}\left(1-\frac{1}{y}\right)(xy-y)+A\mu_{2}S_{2}^{**}\left(1-\frac{1}{z}\right)(1-z)\\ +A\beta_{2}S_{2}^{**}I_{2}^{**}\left(1-\frac{1}{z}\right)(1-zw)+Aa_{21}S_{1}^{**}\left(1-\frac{1}{z}\right)(x-1)\\ +Aa_{12}S_{2}^{**}\left(1-\frac{1}{z}\right)(1-z)+A\beta_{2}S_{2}^{**}I_{2}^{**}\left(1-\frac{1}{w}\right)(zw-w)\\ = & \left(\mu_{1}S_{1}^{**}+a_{21}S_{1}^{**}\right)\left(2-x-\frac{1}{x}\right)+\beta_{1}S_{1}^{**}I_{1}^{**}\left(2-x-\frac{1}{x}\right)\\ +A\left(\mu_{2}S_{2}^{**}+a_{12}S_{2}^{**}\right)\left(2-z-\frac{1}{z}\right)+A\beta_{2}S_{2}^{**}I_{2}^{**}\left(2-z-\frac{1}{z}\right)\\ +a_{12}S_{2}^{**}\left(z-1-\frac{z}{x}+\frac{1}{x}\right)+Aa_{21}S_{1}^{**}\left(x-1-\frac{x}{z}+\frac{1}{z}\right). \end{array}$$

Because of $A=\frac{a_{12}S_{2}^{**}}{a_{21}S_{1}^{**}}$, the above equation can be rewritten down as

$$\begin{array}{rcl} \frac{dU}{dt}{|}_{\left(\text{1}\right)} & = & \left(\mu_{1}S_{1}^{**}+a_{21}S_{1}^{**}+\beta_{1}S_{1}^{**}I_{1}^{**}\right)\left(2-x-\frac{1}{x}\right)\\ +A\left(\mu_{2}S_{2}^{**}+a_{12}S_{2}^{**}+\beta_{2}S_{2}^{**}I_{2}^{**}\right)\left(2-z-\frac{1}{z}\right)\\ +a_{12}S_{2}^{**}\left[\left(z+\frac{1}{z}-2\right)+\left(2-\frac{z}{x}-\frac{x}{z}\right)+\left(x+\frac{1}{x}-2\right)\right]\\ = & \left(\mu_{1}S_{1}^{**}+a_{21}S_{1}^{**}+\beta_{1}S_{1}^{**}I_{1}^{**}-a_{12}S_{2}^{**}\right)\left(2-x-\frac{1}{x}\right)+a_{12}S_{2}^{**}\left(2-\frac{z}{x}-\frac{x}{z}\right)\\ +A\left(\mu_{2}S_{2}^{**}+a_{12}S_{2}^{**}+\beta_{2}S_{2}^{**}I_{2}^{**}-a_{21}S_{1}^{**}\right)\left(2-z-\frac{1}{z}\right). \end{array}$$

Using Eqs. (7) gives rise to

$$\frac{dU}{dt}{|}_{\left(\text{1}\right)}=\Lambda_{1}\left(2-x-\frac{1}{x}\right)+A\Lambda_{2}\left(2-z-\frac{1}{z}\right)+a_{12}S_{2}^{**}\left(2-\frac{z}{x}-\frac{x}{z}\right).$$

The inequality of arithmetic-geometric mean implies $dU/dt{|}_{\left(\text{1}\right)}\leq 0$. The equality holds if and only if x=z=1. That is, when $S_{1}=S_{1}^{**}$ and $S_{2}=S_{2}^{**}$, $dV/dt{|}_{\left(\text{1}\right)}=0$. By using the LaSalle invariant principle [8], the endemic equilibrium $P_{**}$ is globally asymptotically stable. □

**Figure 2:**
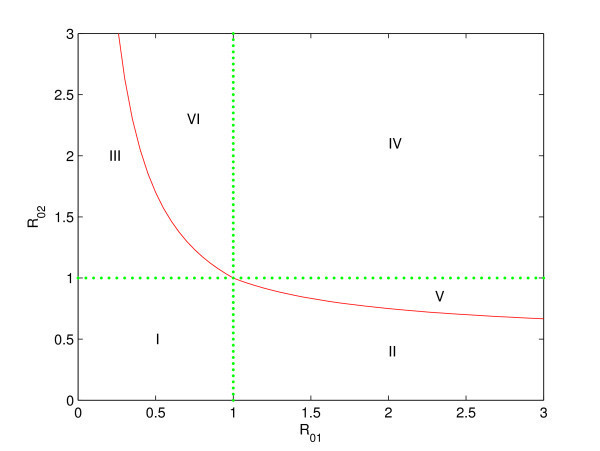
**Bifurcation diagram for system (** **1** **).** In region I, the disease-free equilibrium $P_{0}$ is globally asymptotically stable; in region II, the boundary equilibrium $P_{1*}$ is locally stable; in region III, the boundary equilibrium $P_{2*}$ is locally stable; and in region IV, the endemic equilibrium $P_{**}$ is globally asymptotically stable. In the regions V and VI, the two boundary equilibria are unstable.

Theorems 4.1-4.4 can be summarized in Figure 2. The basic reproduction numbers $R_{01}$ and $R_{02}$ are two important threshold parameters. It shows that if both $R_{01}$ and $R_{02}$ are less than one, the disease-free equilibrium $P_{0}$ is globally asymptotically stable and the disease eventually dies out (the region I in Figure 2); if $R_{01}$ is greater than one, $R_{02}$ is less than one, and $S_{2}^{1*}<S_{2}^{2*}$, the boundary equilibrium $P_{1*}$ is locally stable and the disease persists in patch one but can be eradicated in patch two (the region II in Figure 2); if $R_{02}$ is greater than one, $R_{01}$ is less than one, and $S_{1}^{2*}<S_{1}^{1*}$, the boundary equilibrium $P_{2*}$ is locally stable and the disease persists in patch two but can be eradicated in patch one (the region III in Figure 2); and if $R_{01}>1$, $R_{02}>1$, $S_{2}^{1*}>S_{2}^{2*}$, and $S_{1}^{2*}>S_{1}^{1*}$, there is exactly one endemic equilibrium $P_{**}$, which is globally asymptotically stable by applying Lyapunov method, and the disease persists in two patches (the region IV in Figure 2). In addition, the two boundary equilibria are unstable in the regions V and VI.

## 5 Conclusions and discussions

In this paper, an SIR infectious diseases model with susceptibles dispersal between two disjoint patches has been proposed and analyzed to investigate the impact of susceptibles dispersal on diseases transmission in the whole population. The existence of equilibria is obtained and the basic reproduction numbers $R_{01}$, $R_{02}$, and $R_{0}$ are defined. It is indicated that $R_{01}$ and $R_{02}$ are two important threshold parameters to determine the long-term behavior of the solutions of system (1). The disease-free equilibrium is globally asymptotically stable and the disease ultimately dies out by applying the comparison principle of cooperative systems if the basic reproduction numbers both $R_{01}$ and $R_{02}$ are below unity. The disease persists in patch one and can be eradicated in patch two if $R_{01}$ is above one, $R_{02}$ is below one, and $S_{2}^{1*}<S_{2}^{2*}$. The disease persists in patch two and can be eradicated in patch one if $R_{02}$ is above one, $R_{01}$ is below one, and $S_{1}^{2*}<S_{1}^{1*}$. While the disease uniformly persists in the whole population and the endemic equilibrium is globally asymptotically stable by using the Lyapunov approach if the conditions $R_{01}>1$, $R_{02}>1$, $S_{2}^{1*}>S_{2}^{2*}$, and $S_{1}^{2*}>S_{1}^{1*}$ are satisfied.

System (1) almost shares the same qualitative behavior as the simple SIR epidemic model if dispersal can not be considered in the population. The patchy models need not be considered if only susceptibles disperse among patches. Furthermore, all the patches can be thought of as just one patch and susceptibles dispersal has no influence on disease transmission.

## Authors’ original submitted files for images

Below are the links to the authors’ original submitted files for images. Authors’ original file for figure 1 Authors’ original file for figure 2
