# Supplementary figures and images for: Global dynamics for an SIR patchy model with susceptibles dispersal
Source: Adv Differ Equ. 2012 Aug 1;2012(1):131. doi: 10.1186/1687-1847-2012-131 (PMC7099918; doi:10.1186/1687-1847-2012-131)

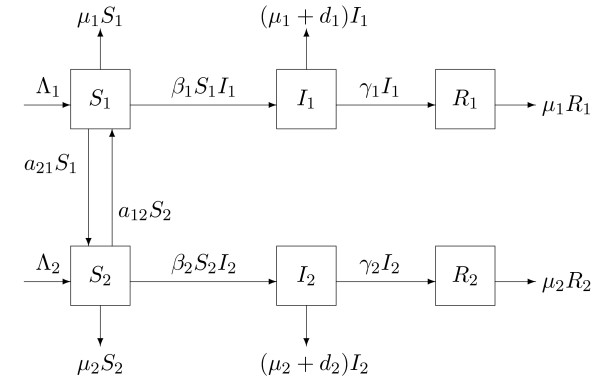

Supplement: Supplementary file 1 — Authors’ original file for figure 1 [file 13662_2012_267_MOESM1_ESM.jpeg]

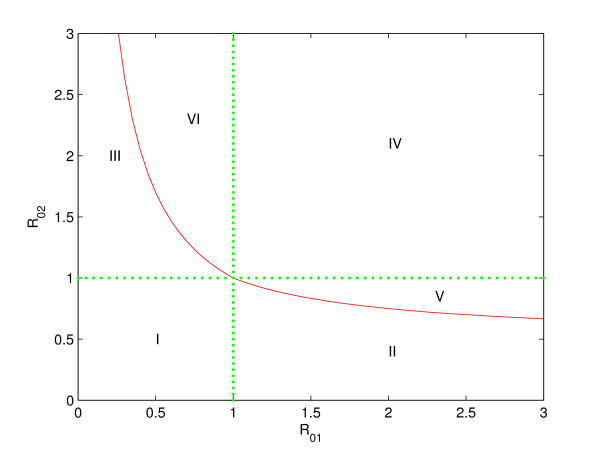

Supplement: Supplementary file 2 — Authors’ original file for figure 2 [file 13662_2012_267_MOESM2_ESM.jpeg]
